# *Ornithoboea brachycarpa* (Gesneriaceae), a new cave-dwelling species from Guangxi, China

**DOI:** 10.3897/phytokeys.276.181524

**Published:** 2026-06-17

**Authors:** Chi Xiong, Jing Wei, Hong Liu, Fang Wen

**Affiliations:** 1 Hubei Provincial Key Laboratory for Protection and Application of Special Plant Germplasm in Wuling Area of China, Key Laboratory of State Ethnic Affairs Commission for Biological Technology, College of Life Sciences, South-Central Minzu University, Wuhan, 430074, Hubei, China College of Life Sciences, South-Central Minzu University Hubei China; 2 Guangxi Key Laboratory of Plant Conservation and Restoration Ecology in Karst Terrain, Guangxi Institute of Botany, Guangxi Zhuang Autonomous Region and Chinese Academy of Sciences, Guilin, 541006, Guangxi, China College of Tourism and Landscape Architecture, Guilin University of Technology Guilin China; 3 College of Tourism and Landscape Architecture, Guilin University of Technology, Guilin, 541006, Guangxi, China Guangxi Institute of Botany, Guangxi Zhuang Autonomous Region and Chinese Academy of Sciences Guilin China; 4 Gesneriad Conservation Center of China (GCCC), National Gesneriaceae Germplasm Resources Bank (NGGRB) of GXIB, Gesneriad Committee of China Wild Plant Conservation Association (GC), Guilin Botanical Garden, Guangxi Zhuang Autonomous Region and Chinese Academy of Sciences, Guilin, 541006, Guangxi, China Guilin Botanical Garden, Guangxi Zhuang Autonomous Region and Chinese Academy of Sciences Guilin China

**Keywords:** Flora of Guangxi, Karst cave, Nanning, new taxon, *Ornithoboea feddei*, taxonomy

## Abstract

*Ornithoboea brachycarpa* C.Xiong, F.Wen & Y.G.Wei, a new cave-dwelling species discovered in Guangxi, China, is described and illustrated. It is morphologically distinctive within the genus by its stamens bearing a beard on the sterile projection, a character not observed in any other known species of *Ornithoboea*. Phylogenetic analysis of ITS and *trn*L-F sequences places it as sister to *O. feddei* (H.Lév.) B.L.Burtt. Currently, only one population comprising approximately 150–200 mature individuals has been confirmed at the type locality. According to the IUCN Red List Categories and Criteria, it is provisionally assessed as Endangered (EN D).

## Introduction

The genus *Ornithoboea* C.B.Clarke (Gesneriaceae) comprises a small group of calcicolous perennial herbs characteristic of karst limestone landscapes throughout Southeast Asia. Its distribution ranges from Peninsular Malaysia and Thailand, through Myanmar, Laos and Vietnam, extending to its northern limit in south China (Middleton and Ly 2008; Scott and Middleton 2014; Middleton et al. 2017). The genus currently includes 17 recognised species and one variety (GRC 2025; POWO 2025). Morphologically, the genus is conspicuous due to its complex floral morphology featuring a prominent palatal beard at the base of the lobes of the lower corolla lip in most species, an annulus of hairs around the mouth of the tube (termed ‘circlet’ in Scott and Middleton (2014)), sterile projections on the stamens in about half of the species, often spirally twisted capsules and calyx lobes that are frequently reflexed in flower and fruit or both. Conversely, pronounced intraspecific variability in leaf morphology presents a significant challenge for species identification (Scott 2013). Molecular phylogenetic evidence (Möller et al. 2009, 2011; Puglisi et al. 2016) has consistently confirmed *Ornithoboea* as a monophyletic genus, placing it as sister to a clade comprising the genera *Kaisupeea* B.L. Burtt and *Rhabdothamnopsis* Hemsl.

In late March 2025, during a botanical survey of the karst cave flora in Shanglin County, Nanning City, Guangxi, China, an unknown species of Gesneriaceae was discovered. The specimens exhibit a caulescent habit with opposite, cordate, serrate leaves and persistent, axillary infructescences from the previous growing season, comprising dehisced capsules with reflexed persistent calyx lobes. However, the absence of flowers at the time precluded definitive identification. Based on the vegetative and fruiting characteristics, the species was tentatively assigned to *Ornithoboea*. A follow-up expedition in early June 2025 found the plants in flower. The presence of genus-specific floral morphology, such as the palatal beard and the circlet, confirmed the specimen’s placement in *Ornithoboea*. Detailed morphological analyses, including *in situ* observations, measurements and floral dissections, were conducted and voucher specimens were collected. Floral dissection revealed that the sterile projections on the stamens bear a distinct yellow beard, a character not observed in any other known species of *Ornithoboea*. This unique feature, together with other morphological differences, strongly suggests its status as an undescribed taxon. To test its placement within the genus, a molecular phylogenetic analysis was conducted using ITS and *trn*L-F sequences. The results placed the new species as sister to *O. feddei* with strong support. The combined evidence supports its formal description as a new species.

## Materials and methods

### Morphological analyses

Morphological descriptions were based on both living field material and herbarium specimens. In total, approximately 30 flowering individuals were observed, measured and photographed *in situ* using digital cameras [Ricoh GR III (Japan, Ricoh Co., Ltd.) and Nikon D7200 (Japan, Nikon Co., Ltd.)]. Additionally, nine voucher specimens were collected, prepared and deposited at the Herbarium of Guangxi Institute of Botany (IBK). Morphological observations of these herbarium specimens were made using an Olympus SZX16 stereomicroscope (Japan, Olympus Co., Ltd.). Descriptive terminology follows Beentje (2016). Herbarium acronyms follow Index Herbariorum (Thiers 2025, continuously updated).

For comparative studies, digital images of additional specimens of closely-related species from Herbaria (E, HTBC, IBK, IBSC, K, KUN, P, PE etc.) were examined via the Chinese Virtual Herbarium (https://www.cvh.ac.cn/) and the Global Biodiversity Information Facility (https://www.gbif.org/). Extensive comparisons were also made against published literature (Middleton and Ly 2008; Scott and Middleton 2014; Middleton et al. 2017; Fu et al. 2022; Wei 2023; Wei et al. 2025).

### Genomic DNA extraction and sequencing

Total genomic DNA was extracted from silica-dried leaves of one sample of the new species collected the type locality (Shanglin County, Guangxi, China) using a modified CTAB method (Chen et al. 2014). DNA samples were sent to Majorbio (Shanghai, China) (http://www.majorbio.com/) for library construction and next-generation sequencing. A paired-end library with an insert size of 350 bp was constructed and sequencing was performed on the Illumina HiSeq 4000 platform with 150 bp paired-end reads. Approximately 1.5 GB of raw reads were generated and subsequently filtered using the FASTX-Toolkit to remove adapters and low-quality reads (http://hannonlab.cshl.edu/fastx_toolkit/download.html). The complete chloroplast genome and the nuclear ribosomal cistron (including the 18S-ITS1-5.8S-ITS2-26S region) were isolated and assembled from the clean reads using GetOrganelle v.1.7.7.0 (Jin et al. 2020).

### Phylogenetic analyses

To determine the phylogenetic placement of the new species, we manually extracted two DNA regions (ITS and *trn*L-F) from the assembled ribosomal DNA and complete plastid genome sequences of the new species (GenBank Acc. ITS: PX606478; *trnL-F*: PX625446). Additional sequences from *Ornithoboea* and related taxa were downloaded from GenBank, based on previous studies (Puglisi et al. 2016; Middleton et al. 2017; Li et al. 2025). The final dataset comprised 29 accessions representing 15 taxa (Table 1). The ingroup included 12 taxa of *Ornithoboea*, while the outgroup consisted of one species of *Rhabdothamnopsis*, and two from *Kaisupeea* (Middleton et al. 2017).

**Table 1.** Voucher information for phylogenetic analyses and GenBank accession numbers.

Taxa	Voucher/Herbarium barcode	Location	ITS	*trn*L-F
*Kaisupeea herbacea* 1	D.J. Middleton et al. 5625 (E)	Thailand	KU203832	KU203927
*Kaisupeea herbacea* 2	D.J. Middleton et al. 5282 (E)	Thailand	KU203831	KU203926
*Kaisupeea herbacea* 3	D.J. Middleton et al. 4518 (E)	Thailand	KU203830	KU203925
*Kaisupeea orthocarpa*	D.J. Middleton et al. 4356 (E)	Thailand	KU203833	KU203928
*Ornithoboea arachnoidea* 1	C. Puglisi et al. LAOS114 (E, SING)	Lao PDR	KY580817	KY580767
*Ornithoboea arachnoidea* 2	C. Puglisi et al. LAOS152 (E, SING)	Lao PDR	KY580818	KY580768
*Ornithoboea arachnoidea* 3	D.J. Middleton et al. 5806 (BKF, E, SING)	Thailand	KY580819	KY580769
*Ornithoboea arachnoidea* 4	D.J. Middleton et al. 4538 (E)	Thailand	JN934751	JN934709
*Ornithoboea arachnoidea* 5	D.J. Middleton et al. 4523 (E)	Thailand	KY580820	KY580770
*Ornithoboea barbanthera*	D.J. Middleton et al. 4257 (BKF, E)	Thailand	KU203839	KU203934
*Ornithoboea brachycarpa*	C.Xiong et al. XC25017 (IBK)	China	PX606478	PX625446
*Ornithoboea feddei*	X.X.Bai et al. XQ 001 (GZAC)	China	PV910737	PV917118
*Ornithoboea flexuosa*	A.R. Rafidah FRI 64358 (KEP)	Malaysia	KU203836	KU203931
*Ornithoboea grandiflora* 1	D.J. Middleton et al. 4975 (BKF, E)	Thailand	KY580821	KY580771
*Ornithoboea grandiflora* 2	D.J. Middleton et al. 5821 (BKF, E, SING)	Thailand	KY580823	KY580773
*Ornithoboea grandiflora* 3	D.J. Middleton et al. 5826 (BKF, E, SING)	Thailand	KY580822	KY580772
*Ornithoboea maxwellii* 1	D.J. Middleton et al. 5815 (BKF, E, SING)	Thailand	KY580825	KY580775
*Ornithoboea maxwellii* 2	D.J. Middleton et al. 5797 (BKF, E, SING)	Thailand	KY580824	KY580774
*Ornithoboea maxwellii* var. minutiflora	D.J. Middleton et al. 5781 (BKF, E, SING)	Thailand	KY580826	KY580776
*Ornithoboea occulta* 1	D.J. Middleton et al. 5702 (BKF, E, SING)	Thailand	KY580827	KY580777
*Ornithoboea occulta* 2	D.J. Middleton et al. 5770 (BKF, E, SING)	Thailand	KY580828	KY580778
*Ornithoboea pseudoflexuosa*	D.J.Middleton et al. 4426 (E, KEP)	Thailand	KY580829	KY580779
*Ornithoboea puglisiae* 1	D.J. Middleton et al. 5617 (BKF, E, P, SING)	Thailand	KU203840	KU203935
*Ornithoboea puglisiae* 2	D.J. Middleton et al. 5814 (BKF)	Thailand	KY580830	KY580780
*Ornithoboea puglisiae* 3	D.J. Middleton et al. 5870 (BKF, SING)	Thailand	KY582831	KY580781
*Ornithoboea puglisiae* 4	P. Triboun 4627 (BK)	Thailand	KY580832	KY580782
*Ornithoboea wildeana* 1	D.J. Middleton et al. 4531 (E)	Thailand	JN934752	JN934710
*Ornithoboea wildeana* 2	D.J. Middleton et al. 5000 (E)	Thailand	KY580833	KY580783
*Rhabdothamnopsis sinensis*	M. Möller & P. Zhou MMO 09–1613 (E)	China	KU203828	KU203923

ITS and *trn*L-F sequences were aligned separately using MAFFT v.7.471 (Katoh and Standley 2013) with default settings, followed by manual adjustment. To assess the combinability of the ITS and *trn*L-F sequences, an incongruence length difference (ILD) test was conducted using PAUP* 4.0 b (Swofford 2002). Aligned regions were concatenated using PhyloSuite v.1.2.3 (Zhang et al. 2020), concatenation being allowed as *P* = 0.12. The best substitution models, GTR+I+G for ITS and GTR+G for *trn*L-F, were selected using ModelFinder v.7.4 (Kalyaanamoorthy et al. 2017) under the corrected Akaike Information Criterion (AICc). A Bayesian Inference (BI) analysis was performed in MrBayes v.3.2.7 (Ronquist et al. 2012) within PhyloSuite v.1.2.3 (Zhang et al. 2020). Two independent runs with four Markov chains for 1,000,000 generations were sampled once every 1000 generations and the convergence of the two independent analyses was deemed to have been achieved when the standard deviation of the splitting frequency was less than 0.01. Posterior probabilities were determined from the posterior distribution after discarding the first 25% trees of each run as burn-in. Finally, the resulting phylogenetic tree was visualised and refined using iTOL version 4 (http://itol.embl.de).

## Results

### Morphological observations

The genus *Ornithoboea* is characterised by several key morphological features: (1) a palatal beard at the base of the lower corolla lip before the division into lobes, which is absent only in *O. maxwellii* (including its variety *O. maxwellii* var. minutiflora); (2) an annulus of hairs (circlet) around the mouth of the corolla tube, present in all known species; (3) sterile projections on the stamens, present in about half of the species (e.g. *O. barbanthera*, *O. flexuosa*, *O. pseudoflexuosa*) and absent in the other half (e.g. *O. feddei*, *O. obovata*, *O. occulta*); (4) calyx lobes reflexed in flower and fruit; and (5) fruit morphology, which varies from spirally twisted to non-twisted. Only three species in the genus possess truly non-twisted fruits: *O. maxwellii*, *O. ovata* and the new species described here, while some other species (e.g. *O. flexuosa*) have weakly or barely twisted fruits.

Examination of *Ornithoboea brachycarpa* reveals that it exhibits the generic features of a palatal beard, a circlet and reflexed calyx lobes and it belongs to the half of the genus that possesses sterile projections on the stamens. However, it is unique within the genus in two respects. First, the sterile projections bear a conspicuous yellow beard; in all other species where sterile projections occur, they are glabrous. Second, its capsules are straight, non-spirally twisted and extremely short (2.5–3 mm long), representing the shortest capsules in the genus. Amongst the other two species with non-twisted fruits (*O. maxwellii* and *O. ovata*), neither possesses a bearded sterile projection. Morphologically, *O. brachycarpa* shows greatest overall similarity to *O. flexuosa* in corolla size, presence of a circlet and palatal beard, short capsules and barely twisted fruits, but the bearded sterile projection readily distinguishes it from these and all other congeners.

### Phylogenetic affinities

**Figure 1.**
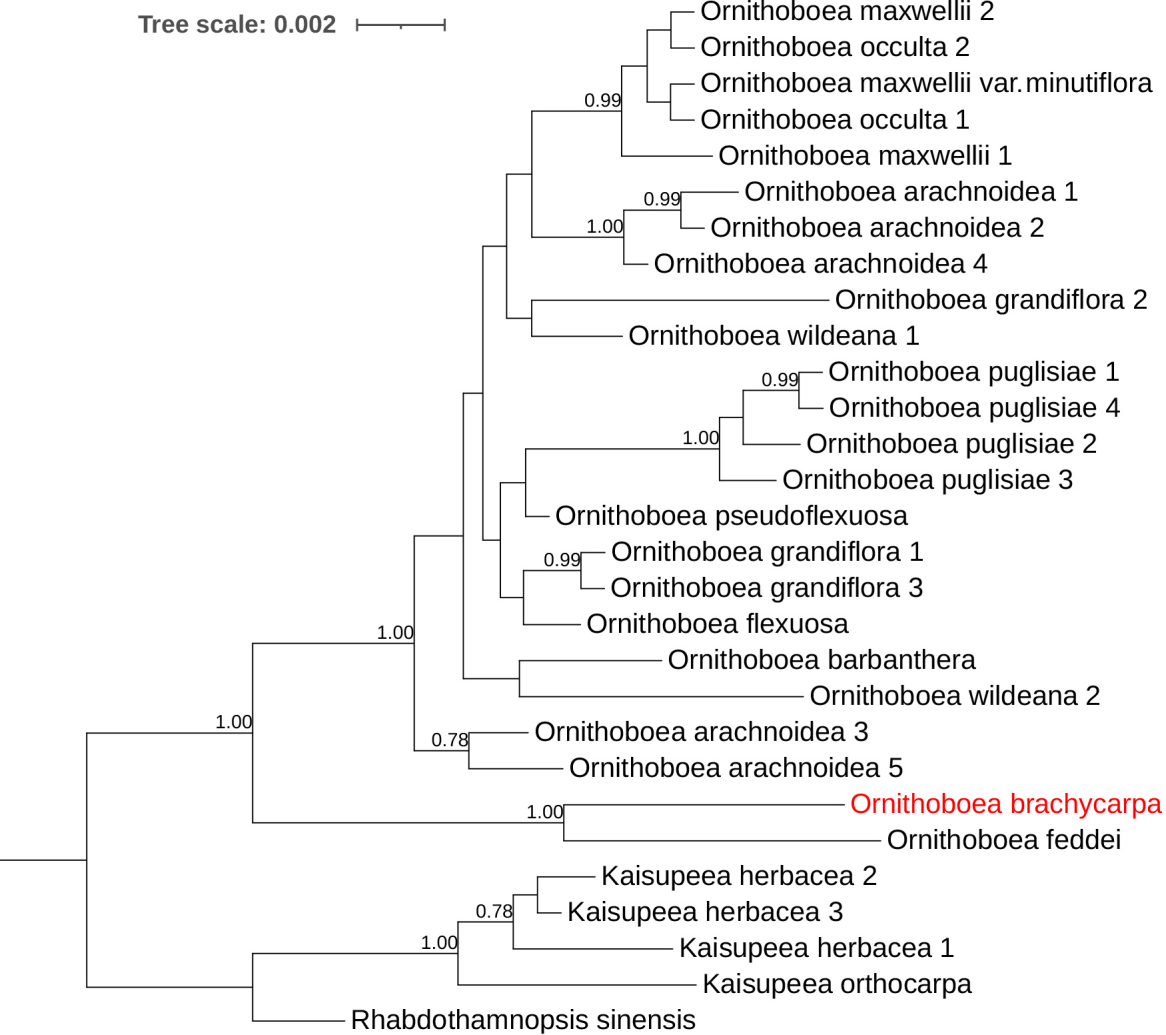
Bayesian tree from analysis of *trn*L-F sequence. The posterior probabilities (PP) of BI are listed at each node (only shown if PP ≥ 0.50). The new species is highlighted in red.

**Figure 2.**
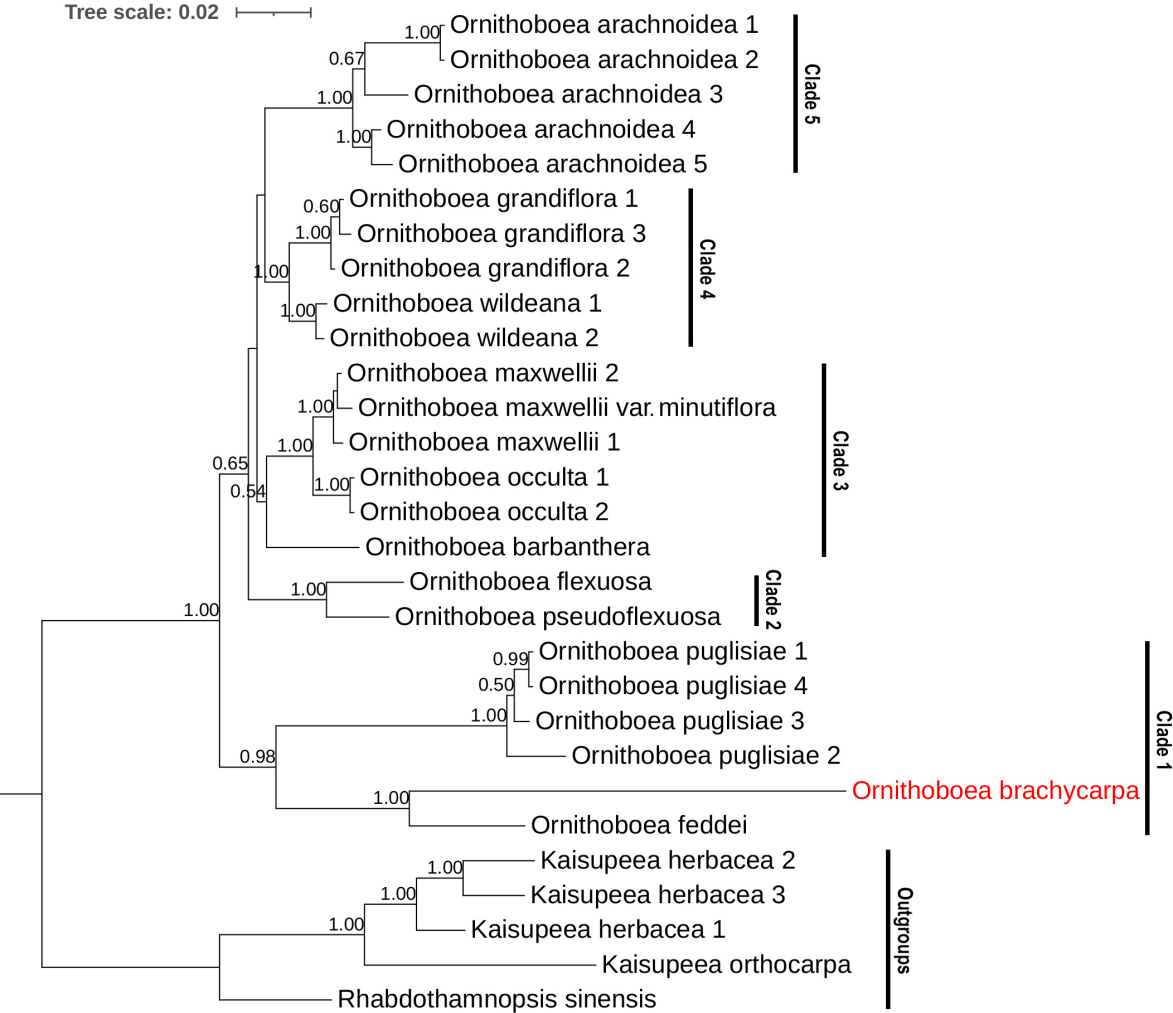
Bayesian tree from analysis of the ITS sequence. The posterior probabilities (PP) of BI are listed at each node (only shown if PP ≥ 0.50). The new species is highlighted in red.

**Figure 3.**
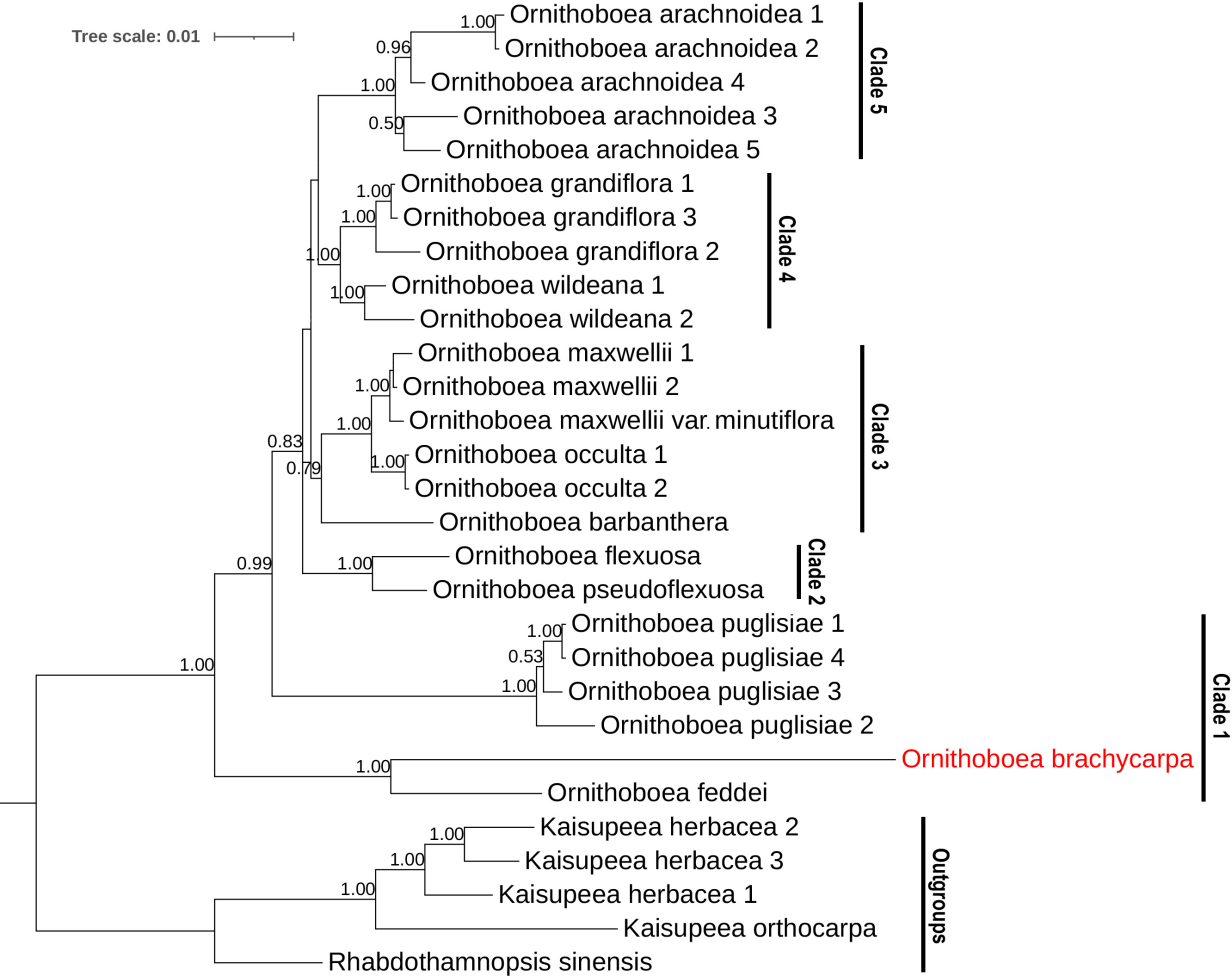
Bayesian tree from analysis of combined ITS and chloroplast *trn*L-F sequences. The posterior probabilities (PP) of BI are listed at each node (only shown if PP ≥ 0.50). The new species is highlighted in red.

The aligned lengths of the ITS and *trn*L-F regions were 676 bp and 881 bp, respectively, yielding a concatenated matrix of 1,557 bp. The Bayesian phylogenetic trees (Figs 1, 2, 3) recovered a strongly supported monophyletic *Ornithoboea* (PP = 1.00) across all three datasets, with the ingroup accessions clearly separated from the outgroup genera *Kaisupeea* and *Rhabdothamnopsis*. Although the *trn*L-F Bayesian tree (Fig. 1) was fully resolved, many nodes were poorly or not supported (PP < 0.50), leading to unresolved relationships within *Ornithoboea* (e.g., the mixing of *O. maxwellii* and *O. occulta*). This is consistent with the typically low phylogenetic signal of this region at the species level.

In contrast, the ITS tree (Fig. 2) resolved all species with multiple samples as monophyletic arranged in five clades. The first diverging clade 1 comprised *O. brachycarpa*, *O. feddei* and *O. puglisiae*, with *O. brachycarpa* and *O. feddei* recovered as sisters with strong support (PP = 1.00). Clade 2 consisted of *O. flexuosa* and *O. pseudoflexuosa* (PP = 1.00). Clade 3 included *O. barbanthera*, *O. occulta*, *O. maxwellii* and *O. maxwellii* var. minutiflora (PP = 0.54). Clade 4 comprised *O. wildeana* and *O. grandiflora* (PP = 1.00). Clade 5 consisted of all accessions of *O. arachnoidea* (PP = 1.00). The analysis of combined ITS and *trn*L-F sequences produced a topology (Fig. 3) largely congruent with the ITS tree, recovering the same five clades with generally improved support values. Notably, *O. brachycarpa* and *O. feddei* were recovered as sisters with maximal support (PP = 1.00) across all three trees.

### Taxonomic treatment

#### Ornithoboea brachycarpa

Taxon classification

Plantae

LamialesGesneriaceae

C.Xiong, F.Wen & Y.G.Wei sp. nov.

602F275C-4C9B-5795-BB09-CFCD8DC9840C

urn:lsid:ipni.org:names:77381676-1

Figs 4, 5

##### Type.

China • Guangxi Zhuangzu Autonomous Region: Nanning City, Shanglin County, Chengtai Township, 23°28'N, 108°45'E, elev. ca. 140 m, 10 June 2025, *Chi Xiong et al. DXGJ250610-01* (holotype IBK!, IBK00472885; isotypes IBK! IBK00472886–00472894).

**Figure 4.**
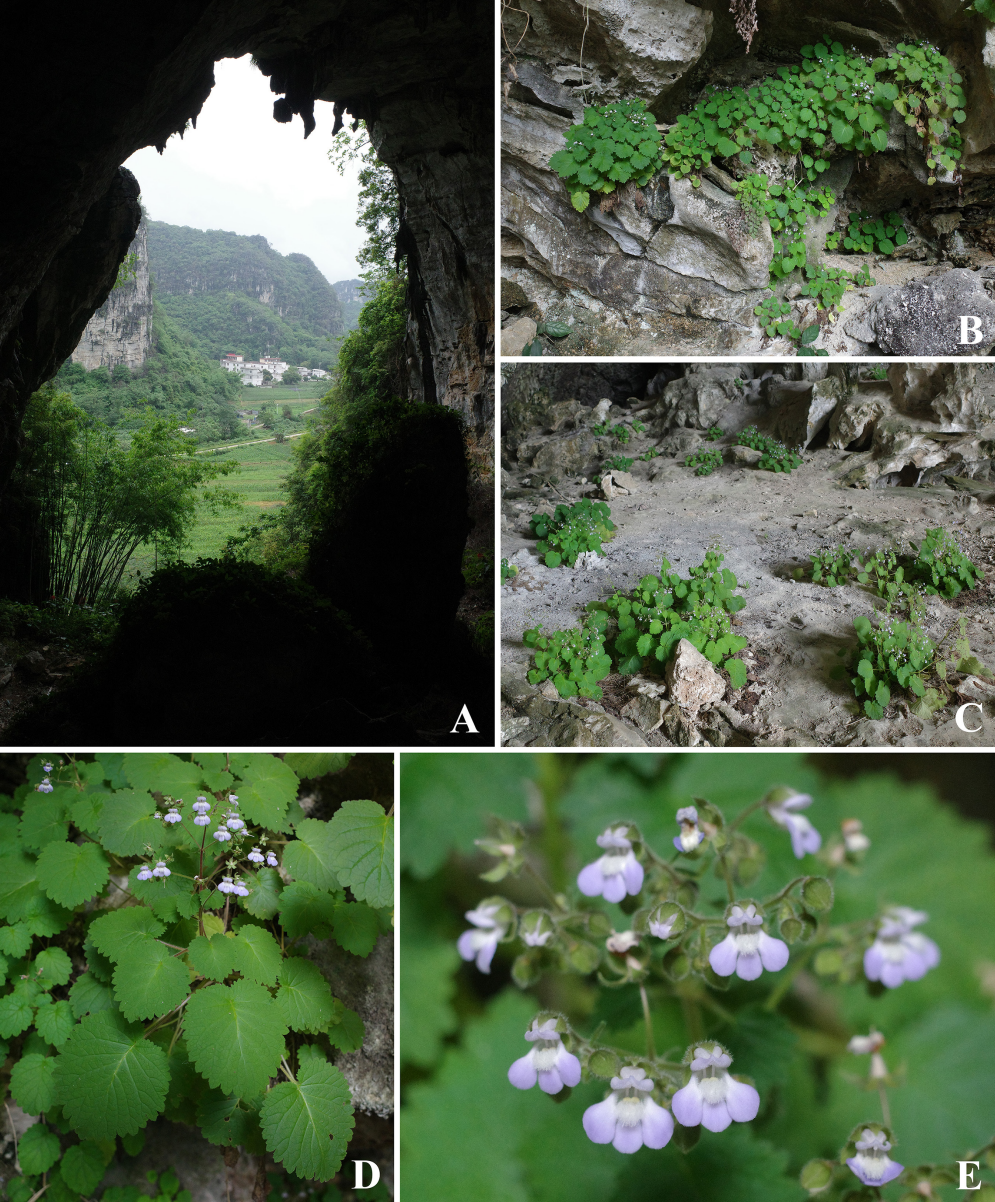
Habitat of *Ornithoboea brachycarpa* C.Xiong, F.Wen & Y.G.Wei, sp. nov. **A–C**. Habitat; **D**. Habit; **E**. Inflorescence (all photographed by Chi Xiong).

**Figure 5.**
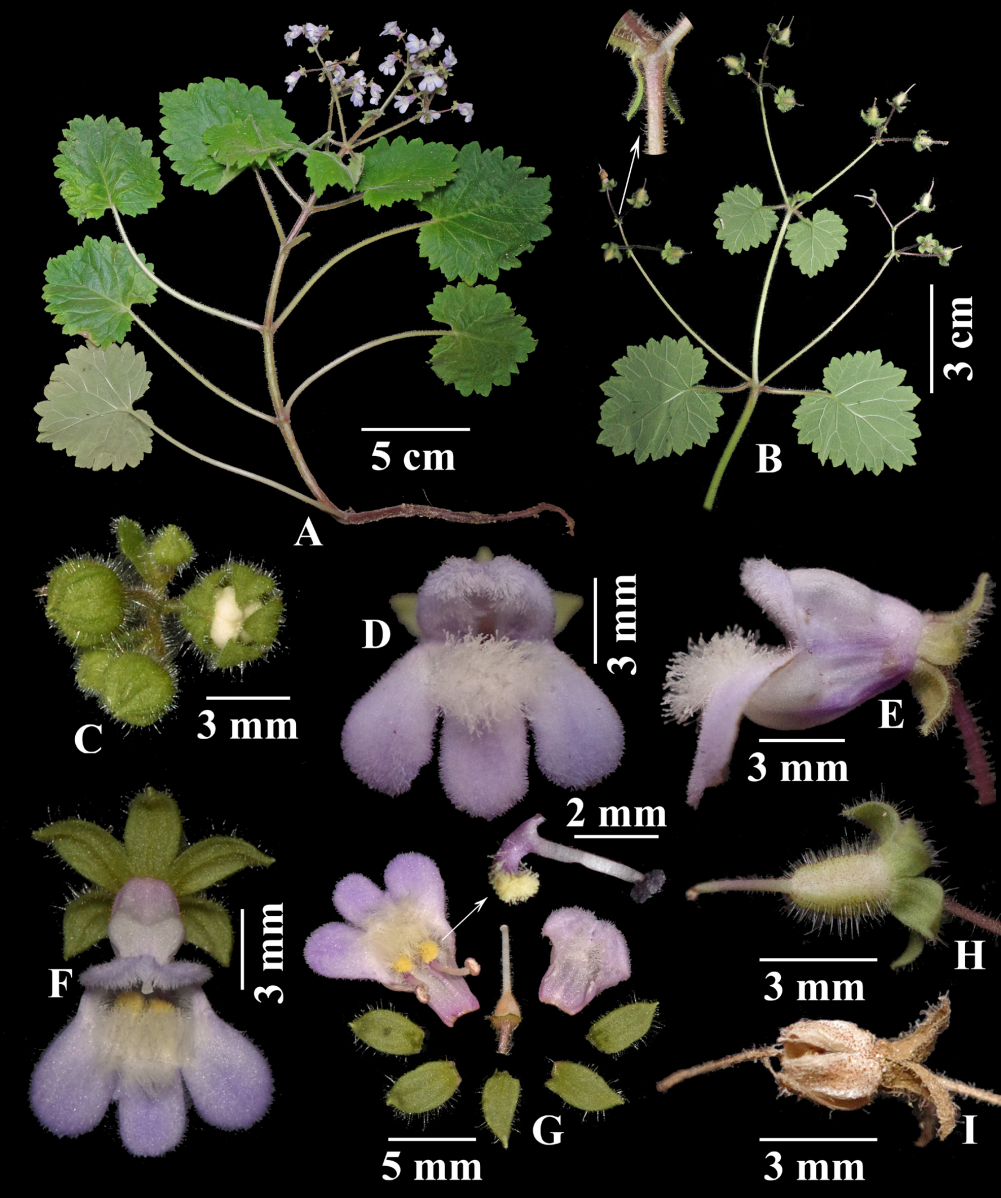
Habitat of *Ornithoboea brachycarpa* C.Xiong, F.Wen & Y.G.Wei, sp. nov. **A**. Habit; **B**. Young infructescence (the arrow shows the bracts); **C**. Flower buds; **D**. Frontal view of flower; **E**. Side view of flower; **F**. Oblique front view of flower; **G**. Dissection of flower (the arrow shows a lateral view of the stamen and sterile projection); **H**. Young fruit; **I**. Mature fruit (all photographed by Chi Xiong).

##### Diagnosis.

*Ornithoboea brachycarpa* is unique in the genus with its bearded sterile projections on the stamens (in species, where present, glabrous) and has also straight, non-spirally twisted, capsules which are rare in the genus.

##### Description.

Biennial to perennial herbs; monocarpic habit, with entire plant senescing after fruit maturation, perennation possible by dormant rhizome buds formed in favourable autumn environmental conditions, leading to new shoots in subsequent spring. ***Stems*** erect to ascending, 20–50 (–75) cm tall, 3.2–5.8 mm in diameter, red at base, densely villous and glandular pubescent throughout; stem internodes 1.2–5.8 cm long. ***Leaves*** opposite, petiolate; petiole 1.8–13.7 cm long, 2–4 mm in diameter, light green, base sometimes red, densely glandular puberulous; leaf blade suborbicular to ovate, 3.2–9.7 × 2.8–8.6 cm, thinly chartaceous, adaxially green, abaxially pale green, densely white-glandular puberulous on both surfaces, base slightly unequal, cordate, margin irregularly denticulate to crenate, apex acute, obtuse to rounded, secondary veins 3–6 pairs, tertiary venation reticulate. ***Inflorescence*** terminal or subterminal, 5–18 cm long, densely glandular puberulous, peduncle 2.5–12 cm long, 1–2 mm in diameter; bracts 2, opposite, free, lanceolate, 4.2–5.8 × 1–1.2 mm, pubescent on both sides; pedicels 8–18 mm long, ca. 1 mm in diameter. ***Calyx*** 5-parted to base, lobes ovate to elliptic, green, 3-veined, 4–4.5 × 2–2.4 mm, pubescent on both sides, margin entire, apex acute, reflexed in flowering and fruiting. ***Corolla*** bilabiate, 8–10 mm long, light purple to violet, sparsely glandular puberulous outside; tube 3–3.5 mm long, inflated in side view, slightly compressed ventrally, inside light purple; upper lip slightly 2-lobed, erect to slightly reflexed, 1.5–2 mm long, densely glandular-pubescent with circlet of longer hairs, lobes ca. 0.5 mm long, apex rounded; lower lip 3-lobed, slightly reflexed, ca. 4.5 mm long, pubescent in addition to the palatal beard at base of lobes, lobes slightly obovate, 3.4–4.2 × 2.6–3.2 mm, apex rounded. ***Stamens*** 2; filaments ca. 3 mm long, adnate ca. 3.5 mm from corolla base and bent backwards to base of tube with a distinct sterile projection bearing yellow beard, projection ca. 1 mm long, 0.4–0.6 in diameter, beard 0.3–0.5 mm long; anthers turning dark purple when dehisced, reniform, 1 × 0.5 mm, slightly constricted at middle, with the dehiscence slit, white; staminodes 3, glabrous, white, lateral ones linear, incurved, ca. 1.5 mm long, adnate ca. 1 mm above corolla tube base, the central one inconspicuous, adnate ca. 0.8 mm above corolla tube base. ***Pistil*** 5–6 mm long; ***ovary*** 1.2–1.5 mm long, white, glandular pubescent, with orange glands; ***style*** ca. 3.5 cm long, ca. 0.5 mm in diameter, white, sparsely glandular-pubescent; stigma capitate. ***Immature capsule*** 2–2.5 mm long, pale green, glandular pubescent, with orange glands; ***mature capsule*** straight, 2.5–3 mm long, ca. 1.5 mm in diameter, yellowish-brown, non-spirally twisted, style and orange glands persistent, longitudinally dehiscing into four valves.

##### Phenology.

Flowering from May to June; fruiting from June to September.

##### Etymology.

The specific epithet ‘brachycarpa’ is derived from the Greek words ‘*brachys*’ (short) and ‘*karpos*’ (fruit), referring to its having the shortest fruits within the genus *Ornithoboea*. The name and concept of this species were established jointly by C.Xiong, F.Wen & Y.G.Wei, with Prof. Y.G. Wei contributing his expertise in Chinese Gesneriaceae and cave flora.

##### Vernacular name.

guǎng xī xǐ què jǔ tái (Chinese pronunciation); 广西喜鹊苣苔(Chinese name).

##### Distribution and ecology.

This new species is currently known only from the type locality at an elevation of ca. 140 m. It grows on dry rock surfaces at the entrance or on the ground inside a karst cave (Fig. 4A–D). The most frequent co-occurring vascular plant species include *Ficus tinctoria* subsp. *gibbosa* (Blume) Corner (Moraceae), *Pteris vittata* L. and *Adiantum capillus-veneris* L. (Pteridaceae).

##### Conservation status.

*Ornithoboea brachycarpa* is currently known from only one population at the type locality. The population size is estimated at around 150–200 mature individuals. The species inhabits the entrance and ground inside a karst cave located on a hillside near a village. The cave shows clear signs of human activity, with ritual burial jars stored inside as part of a local traditional cave‐burial custom. Occasional visits by villagers for ceremonial practices, including tomb-sweeping during the Qingming Festival, involve the removal of surrounding plants. Although not intentionally targeted, individuals of *O. brachycarpa* may be damaged or removed during these activities, exposing the population to potential disturbance. Following the IUCN guidelines (IUCN 2024), the species is provisionally assessed as Endangered (EN D).

## Discussion

*Ornithoboea brachycarpa* is morphologically highly distinctive within the genus. The presence of a beard on the sterile projection on the stamens is a discrete, autapomorphic character not observed in any other known species of *Ornithoboea* (where sterile projections, when present, are glabrous). Morphologically, the new species shows greatest overall similarity to *O. flexuosa* in terms of corolla size, presence of a circlet on the upper lip, a palatal beard on the lower lip, short capsules and barely twisted fruits. Given that *O. flexuosa* is also known to inhabit karst caves, the morphological similarity between these species may reflect convergent evolution under similar cave-dwelling selective pressures, since the two species are phylogenetically not closely related (Figs 1, 2, 3).

Based on our molecular analyses of ITS and t*rn*L-F sequences (Figs 1, 2, 3), *O. brachycarpa* is resolved as sister to *O. feddei* with strong support. Despite this, the morphological differences between the two species are striking. *Ornithoboea feddei* lacks sterile projections, possesses twisted fruits (6.3–14 mm long) and has a longer corolla tube (4–6.5 mm) and larger floral lobes (see Table 2 for a full comparison). These marked differences suggest that the phylogenetic proximity does not reflect morphological similarity and the distinct traits of *O. brachycarpa* — particularly the bearded sterile projection — may have arisen as adaptations to its specific cave-dwelling habitat. However, it should also be noted that this phylogenetic placement may be an artefact affected by long branches, particularly for *O. brachycarpa*. Notably, our results differ from those of Middleton et al. (2017), in which *O. puglisiae* and *O. arachnoidea* were recovered as sisters, whereas in our trees, these two species are placed in distantly related clades. Further sampling and the use of additional molecular markers may help resolve these relationships more robustly.

**Table 2.** Morphological and ecological comparison of *Ornithoboea brachycarpa*, *O. flexuosa* and *O. feddei*.

Characters	*Ornithoboea brachycarpa*	*O. flexuosa*	*O. feddei*
Leaf-blade	suborbicular to ovate, margin irregularly denticulate to crenate, base slightly unequal, cordate, apex acute, obtuse to rounded	ovate to elliptic, margin weakly to strongly crenate, bicrenate to dentate or duplicato-dentate, base unequal, oblique to rounded, apex acute to broadly acute	broadly to narrowly ovate, margin crenate or bicrenate, base occasionally oblique, slightly cordate, apex acute to narrowly acute
Secondary veins	3–6 pairs	8–10 pairs	9–10 pairs
Calyx lobes	ovate to elliptic, 3–4.5 mm long, apex acute	ovate, 4.5–5.5 mm long, apex narrowly acute	lanceolate to oblong-lanceolate, 4.5–10 mm long, apex narrowly acute
Corolla	8–10 mm long, light purple to violet, tube 3–3.5 mm long	9.5–10 mm long, purple or lilac, tube 3.2–4 mm long	9–15.5 mm long, light blue, tube 4–6.5 mm long
Upper lip	slightly 2-lobed, lobes ca. 0.5 mm long, apex rounded, densely glandular-pubescent with circlet of longer hairs	slightly 2-lobed, lobes ca. 0.7 mm long, notched in centre, circlet of short white hairs	2-lobed, lobes 2–3.5 mm long, apex retuse, circlet of short white hairs
Lower lip	ca. 4.5 mm long, lobes slightly obovate, 3.4–4.2 × 2.6–3.2 mm, apex rounded	ca. 7 mm long, slightly reflexed, lobes 3–4 × 1.3–1.6(–2.5) mm, apex narrowly rounded to acute	5.5–9.1 mm long, lobes orbicular, 2–3.5 × 1.8–2.3 mm, apex broadly pyramidal
Stamens	with a distinct sterile projection bearing yellow beard	with a distinct glabrous sterile projection	without pronounced sterile projection
Fruit	2.5–3 × ca. 1.5 mm, non-spirally twisted	4.3–7(–8.2) × 1.6–2.5 mm, barely twisted by quarter turn to non-twisted	6.3–14 × 2.3–2.7 mm, twisted
Habitat	cave-dwelling	both	non-cave-dwelling

**Figure 6.**
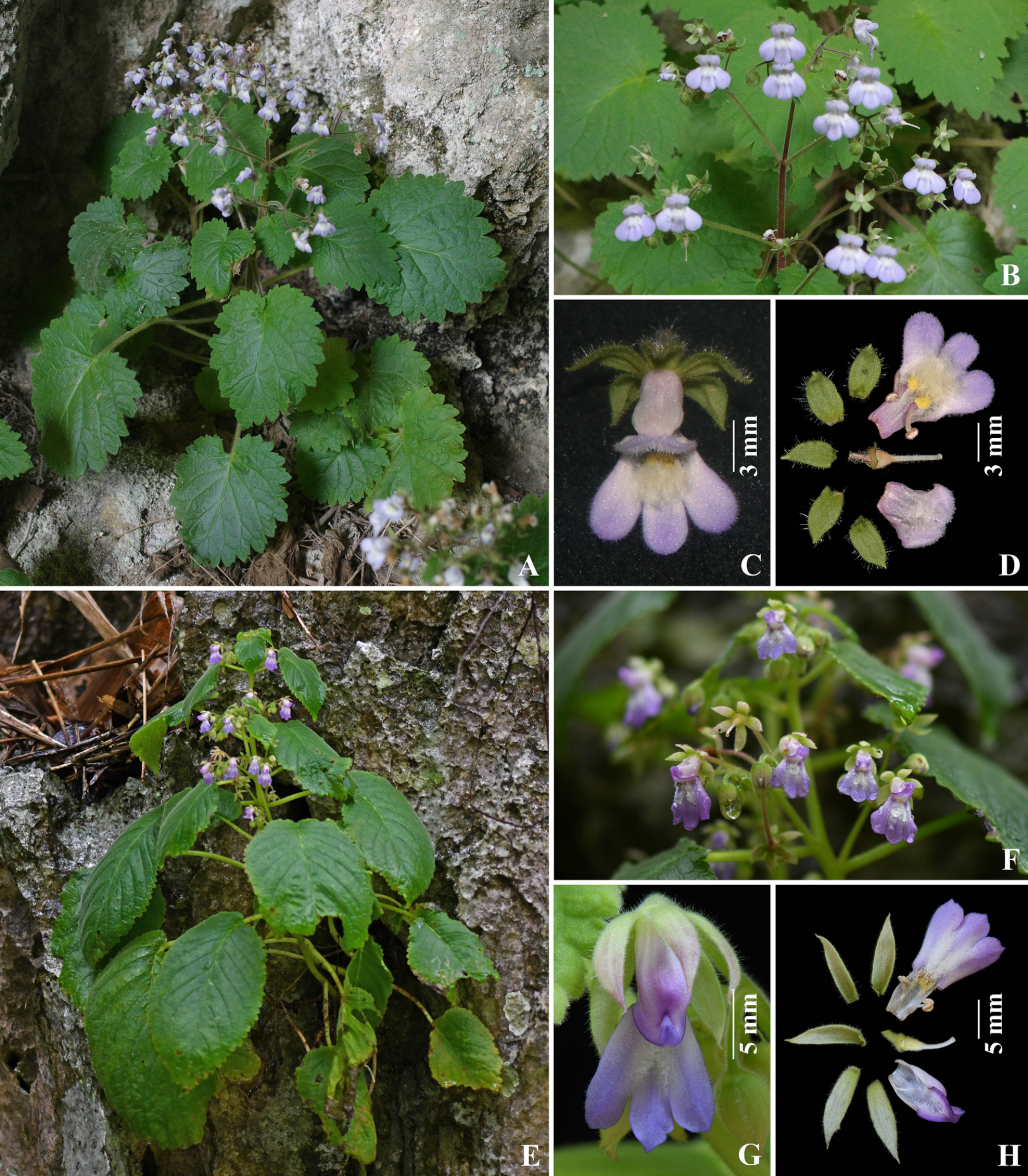
*Ornithoboea brachycarpa* (A–D) and *O. feddei* (E–H). **A, E**. Plant in natural habitat; **B, F**. Inflorescence; **C, G**. Oblique front view of flower; **D, H**. Dissection of flower (**A–D** photographed by Chi Xiong, **E–H** by Xin-Xiang Bai).

The functional significance of the bearded sterile projection in *O. brachycarpa* warrants further consideration. Staminal structures in other plant groups, such as the lever mechanism in *Salvia* (Claßen-Bockhoff et al. 2003), are known to enable precise pollen deposition on pollinators. Whether a similar functional differentiation exists in *Ornithoboea* — and whether the bearded projection plays any role in pollinator attraction or positioning — remains unknown. The yellow beard could potentially mimic fertile anthers, guiding pollinators to a specific position within the flower, but this hypothesis requires testing. Given that the flower lacks a nectar disc, a deceptive pollination strategy is plausible. Future field observations of pollinator behaviour, including whether the sterile projection is physically contacted during foraging, are needed to clarify the functional significance of this structure.

## Supplementary Material

XML Treatment for Ornithoboea brachycarpa

## Additional information

### Artificial Intelligence (AI) use

The authors accept full responsibility for the content of the manuscript, including the disclosure of any use of AI.

No AI tools were used in the preparation of this manuscript.

### Author ORCIDs

C. Xiong https://orcid.org/0000-0002-9082-2539

J. Wei https://orcid.org/0009-0004-7378-1044

H. Liu https://orcid.org/0000-0001-7227-2476

F. Wen https://orcid.org/0000-0002-3889-8835

### Data availability

All of the data that support the findings of this study are available in the main text or Supplementary Information.

## Supplementary materials

Supplementary material 1 Sequence alignment file 1 Data type fasta https://binary.pensoft.net/file/1682501This dataset is made available under the Open Database License (http://opendatacommons.org/licenses/odbl/1.0/). The Open Database License (ODbL) is a license agreement intended to allow users to freely share, modify, and use this Dataset while maintaining this same freedom for others, provided that the original source and author(s) are credited. Chi Xiong, Jing Wei, Hong Liu, Fang Wen

Supplementary material 2 Sequence alignment file 2 Data type fasta https://binary.pensoft.net/file/1682502This dataset is made available under the Open Database License (http://opendatacommons.org/licenses/odbl/1.0/). The Open Database License (ODbL) is a license agreement intended to allow users to freely share, modify, and use this Dataset while maintaining this same freedom for others, provided that the original source and author(s) are credited. Chi Xiong, Jing Wei, Hong Liu, Fang Wen
